# Curcumin Sensitizes Hepatocellular Carcinoma Cells to Radiation via Suppression of Radiation-Induced NF-*κ*B Activity

**DOI:** 10.1155/2015/363671

**Published:** 2015-10-11

**Authors:** Fei-Ting Hsu, Yu-Chang Liu, Tsu-Te Liu, Jeng-Jong Hwang

**Affiliations:** ^1^Department of Biomedical Imaging and Radiological Sciences, National Yang-Ming University, Taipei 112, Taiwan; ^2^Department of Medical Imaging, Taipei Medical University Hospital, Taipei 110, Taiwan; ^3^Department of Radiation Oncology, National Yang-Ming University Hospital, Yilan 260, Taiwan; ^4^Division of Gastroenterology, Department of Internal Medicine, National Yang-Ming University Hospital, Yilan 260, Taiwan

## Abstract

The effects and possible underlying mechanism of curcumin combined with radiation in human hepatocellular carcinoma (HCC) cells *in vitro* were evaluated. The effects of curcumin, radiation, and combination of both on cell viability, apoptosis, NF-*κ*B activation, and expressions of NF-*κ*B downstream effector proteins were investigated with 3-(4,5-dimethylthiazol-2-yl)-2,5-diphenyltetrazolium bromide (MTT), NF-*κ*B reporter gene, mitochondrial membrane potential (MMP), electrophoretic mobility shift (EMSA), and Western blot assays in Huh7-NF-*κ*B-*luc2*, Hep3B, and HepG2 cells. Effect of I kappa B alpha mutant (I*κ*B*α*M) vector, a specific inhibitor of NF-*κ*B activation, on radiation-induced loss of MMP was also evaluated. Results show that curcumin not only significantly enhances radiation-induced cytotoxicity and depletion of MMP but inhibits radiation-induced NF-*κ*B activity and expressions of NF-*κ*B downstream proteins in HCC cells. I*κ*B*α*M vector also shows similar effects. In conclusion, we suggest that curcumin augments anticancer effects of radiation via the suppression of NF-*κ*B activation.

## 1. Introduction

Hepatocellular carcinoma (HCC) is ranked the fifth most common malignancy and the third leading cause of cancer-related mortality worldwide [1]. Curative treatment strategies including surgery, liver transplantation, and local ablative therapy are unsuitable for patients with advanced HCC [2]. Radiotherapy, despite being not the main treatment modality for advanced HCC, may have palliative effect and increase survival of patients. Most patients have local recurrence after irradiation and tumor dose escalation may be the key to successful treatment [3]. However, dose escalation of radiotherapy in HCC is limited by the normal liver tolerance to radiation. Recent advances in radiotherapy technology have provided precise radiation dose delivery to tumor site while avoiding normal liver irradiation [4]. Meanwhile, it is also critical to find radiosensitizer for HCC which can yield more tumoricidal efficacy without escalating radiation dose level.

Activation of transcription factor NF-*κ*B is frequently found in several kinds of cancer cells and related to aggressive tumor growth and chemo- and radioresistance during cancer treatment [5]. It has been shown that most chemotherapeutic agents and radiation therapy could activate NF-*κ*B in cancer cells both* in vitro* and* in vivo* [6]. A number of genes that are linked to NF-*κ*B, including MMP-9, VEGF, cyclin-D1, Bcl-2, and XIAP, contribute to the development of tumor radioresistance [5–7]. Meanwhile, constitutive activation of NF-*κ*B has been observed in liver cancer cells but not in normal liver tissues [8]. Some retrospective studies indicate that overexpressions of NF-*κ*B downstream effector proteins (e.g., VEGF, MMP-9, cyclin-D1, and XIAP) in cancer cells portend poorer survival for HCC patients [9, 10]. In addition, preclinical studies reveal that inhibition of NF-*κ*B signaling cascade may sensitize various cancer cells including oral squamous cell carcinoma, HCC, colorectal cancer, and breast cancer to radiotherapy [11–14].

Curcumin, a phytochemical compound extracted from Curcuma longa, is well known for its anti-inflammatory and anticancer effects. Curcumin is also known as NF-*κ*B inhibitor to reduce tumor growth, angiogenesis, and tumor metastasis via suppression of NF-*κ*B-regulated effector proteins [15]. In our previous study, we used curcumin as radiosensitizer to synergistically enhance the radiosensitivity of oral cancer via the suppression of radiation-induced NF-*κ*B activity [11]. Curcumin combines radiation which has similar effect in other cancer cells such as colorectal and prostate cancers [16, 17]. Nevertheless, whether curcumin has radiosensitization effect through NF-*κ*B inhibition in HCC cells remains to be elucidated. The effects of treatments with curcumin, radiation, and combination of both on cell viability, apoptosis, NF-*κ*B activity, and expressions of NF-*κ*B-regulated proteins in Huh-7/NF-*κ*B-*luc2*, Hep3B, and HepG2 were investigated. The results demonstrate that curcumin could sensitize HCC to radiation via suppression of NF-*κ*B activation and suggest that the combination of curcumin and radiation has therapeutic potential for HCC patients.

## 2. Materials and Methods

### 2.1. Cell Culture

Three human hepatocellular carcinoma cell lines including Huh7, Hep3B, and HepG2 cells were used in this study. Huh7 cell line was kindly provided by Dr. Jason Chia-Hsien Cheng at the Department of Radiation Oncology, National Taiwan University Hospital, Taipei, Taiwan. Hep3B and HepG2 cell lines were purchased from the American Type Culture Collection (ATCC, Gaithersburg, MD, USA). All cell lines were maintained in Dulbecco's Modified Eagle's Medium (DMEM) with supplemental 10% fetal bovine serum (FBS) and 0.5% penicillin/streptomycin (PS) and cultured at 37°C in a humidified incubator containing 5% CO_2_. The Huh7/NF-*κ*B-*luc2* stable clone was maintained in the same medium with additional 500 *μ*g/mL of G418 (Calbiochem, Darmstadt, Hesse, Germany).

### 2.2. Construction of NF-*κ*B/*luc2* Vector

The NF-*κ*B-responsive sequence was isolated from pNF-*κ*B-*luc* (Clontech, Mountain View, CA, USA) and inserted into pGL4-*luc2 *(Promega, Madison, WI, USA), resulting in a pNF-*κ*B-*luc2*. The procedure of vector construction was described in detail previously [18].

### 2.3. Plasmid Transfection and Stable Clone Selection

Huh7 cells were transfected with pNF-*κ*B-*luc2* by using jetPEI transfection reagent (Polyplus Transfection, Strasbourg, Alsace, France). Huh7/NF-*κ*B-*luc2* stable clone was established as previously described [18]. I*κ*B*α* mutant vector (p-I*κ*B*α*M, Clontech, Mountain View, CA, USA) was used for the inhibition of NF-*κ*B activation in Huh7/NF-*κ*B-*luc2 *cells. The procedure of p-I*κ*B*α*M transfection in Huh7/NF-*κ*B-*luc2 *cells was described in detail previously [18].

### 2.4. Irradiation

X-ray irradiator (RS 2000; Rad Source Technologies, Suwanee, GA, USA) was used for radiation exposure and was executed with the following parameters: 1.03 Gy/min; 80 cm source-to-skin distance (SSD); field size 30 × 30 cm^2^.

### 2.5. 3-(4,5-Dimethylthiazol-2-yl)-2,5-diphenyltetrazolium Bromide (MTT) Assay

Cells of three human HCC cell lines (Huh7/NF-*κ*B-*luc2*, Hep3B, and HepG2) were seeded into 96-well plates at a density of 3 × 10^4^ cells/well and cultured for 24 h. The treatment conditions for HCC cells in different groups were described in detail in figure legends. Cell viability was evaluated by MTT assay with the same protocol as described previously [19].

### 2.6. NF-*κ*B Luciferase Reporter Gene Assay

Huh7/NF-*κ*B-*luc2 *cells were seeded into 96-well plates at a density of 3 × 10^4^ cells/well and cultured for 24 h. The treatment conditions for HCC cells in different groups were described in detail in figure legends. D-luciferin (100 *μ*L of 500 *μ*M, Xenogen) was added to each well, and detection of photon signal was acquired for 1 min using an IVIS50 Imaging System (Xenogen, Hopkinton, MA, USA). The calculation of Relative NF-*κ*B activity was based on ratio of ROI value of treatment group and ROI value of control group to be normalized by cell viability of treatment group [19].

### 2.7. Western Blotting

2 × 10^6^ cells were seeded in 10 cm diameter dishes and incubated for 24 h. The treatment conditions for HCC cells in different groups were described in detail in figure legends. Total protein was extracted from cells in different treatments with lysis buffer (50 mM Tris-HCl pH 8.0, 120 mM NaCl, 0.5% NP-40, and 1 mM phenylmethanesulfonyl fluoride). The protein levels of MMP-9, VEGF, XIAP, Bcl-2, and cyclin-D1 were assayed with Western blot as described previously [19]. XIAP was purchased from Abcam (Cambridge, UK). Other reagents and antibodies were purchased from Merck Millipore (Darmstadt, Germany).

### 2.8. Electrophoretic Mobility Shift Assay (EMSA)

2 × 10^6^ HCC cell lines (Hep3B and HepG2) were seeded into 10 cm diameter dishes and incubated for 24 h prior to the treatment with 30 *μ*M curcumin for 24 h, 10 Gy irradiation, and combination of both, respectively. Nuclear extraction kit (Chemicon, Temecula, CA, USA) was used to collect the nuclear fractions of HCC cells following the manufacturer's protocol. The following DNA sequences were synthesized for EMSA analysis: sense: AGTTGAGGGGACTTTCCCAGGC and antisense: GCCTGGGAAAGTCCCCTCAACT. The NF-*κ*B/DNA binding activity was determined with LightShift Chemiluminescent EMSA kit (Thermo Scientific, Rockford, IL, USA). The detailed procedure of EMSA was described previously [18].

### 2.9. Detection of Mitochondrial Membrane Potential (ΔΨ_*m*_)

2 × 10^5^ cells were seeded in 12-well plates and incubated for 24 h. The treatment conditions for HCC cells in different groups were described in detail in figure legends. After treatment, cells were collected with centrifugation and washed with PBS, then resuspended in 500 *μ*L of 4 *μ*M DiOC6, and incubated at 37°C for 30 min and then analyzed by flow cytometry [20].

### 2.10. Statistical Analysis

All data are shown as means ± standard errors. Statistical analysis was performed using Student's *t*-test. Differences between the means were considered significant if *P* < 0.05.

## 3. Results

### 3.1. Radiation Induces NF-*κ*B Activity and Increases Expressions of NF-*κ*B Downstream Effector Proteins in Huh7/NF-*κ*B-*luc2* Cells

**Figure 1:**
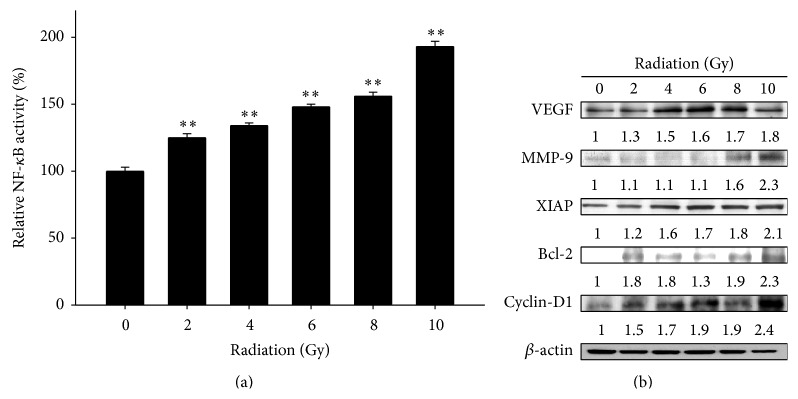
Effects of radiation on NF-*κ*B activation and expressions of NF-*κ*B downstream effector proteins in Huh7/NF-*κ*B-*luc2* cells. Cells were irradiated with various radiation doses (0–10 Gy) and then incubated for 24 h. (a) NF-*κ*B activation was evaluated with IVIS50 optical imaging system. ^*∗∗*^ *P* < 0.01 compared with that of the control. (b) Expressions of NF-*κ*B downstream effector proteins (i.e., VEGF, MMP-9, XIAP, Bcl-2, and cyclin-D1) assayed with Western blot are increased in a dose-dependent manner.

Cells were irradiated with different doses (0–10 Gy) and incubated for 24 h. Radiation significantly induced NF-*κ*B activity in a dose-dependent manner (Figure 1(a)). Cells irradiated with 10 Gy showed maximal NF-*κ*B activity and were selected for subsequent experiments. Radiation also increased NF-*κ*B downstream effector proteins expressions including VEGF, MMP-9, XIAP, BCL-2, and cyclin-D1 (Figure 1(b)).

### 3.2. Curcumin Inhibits Tumor Cell Proliferation and Suppresses Expressions of NF-*κ*B Downstream Effector Proteins in Huh7/NF-*κ*B-*luc2* Cells

**Figure 2:**
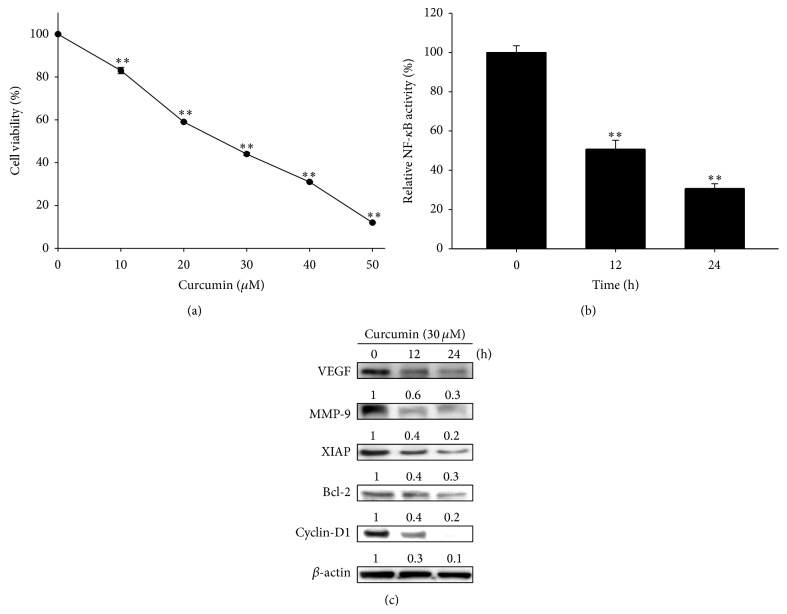
Effects of curcumin on cell viability, NF-*κ*B activation, and expressions of NF-*κ*B downstream effector proteins in Huh7/NF-*κ*B-*luc2* cells. (a) Cells were treated with different concentration (0–50 *μ*M) of curcumin for 24 h. Cell viability was evaluated with MTT assay. ^*∗∗*^ *P* < 0.01 compared with that of the control. (b) Cells were treated with 30 *μ*M curcumin for 0, 12, and 24 h. NF-*κ*B activation was assayed by bioluminescent imaging. ^*∗∗*^ *P* < 0.01 compared with control group. (c) Cells were treated with 30 *μ*M curcumin for 0, 12, and 24 h. Expressions of NF-*κ*B downstream effector proteins (i.e., VEGF, MMP-9, XIAP, BCL-2, and Cyclin-D1) assayed with Western blot are decreased with increase in treatment time.

Figure 2(a) shows that cell viability is significantly decreased by curcumin in a dose-dependent manner (0–50 *μ*M). Fifty percentage of Huh7/*NF-κB-luc2* cells viability was defined as IC_50_ in this study. The IC_50_ for curcumin is approximately 30 *μ*M according to the result of MTT assay and is used for the following experiments. Curcumin significantly inhibits the activation of NF-*κ*B (Figure 2(b)) and suppresses the expressions of NF-*κ*B downstream effector proteins including VEGF, MMP-9, XIAP, Bcl-2, and cyclin-D1 in a time-dependent manner (Figure 2(c)).

### 3.3. Curcumin Sensitizes HCC Cells to Radiation and Inhibits Radiation-Induced NF-*κ*B Activity and Expressions of NF-*κ*B Downstream Effector Proteins

**Figure 3:**
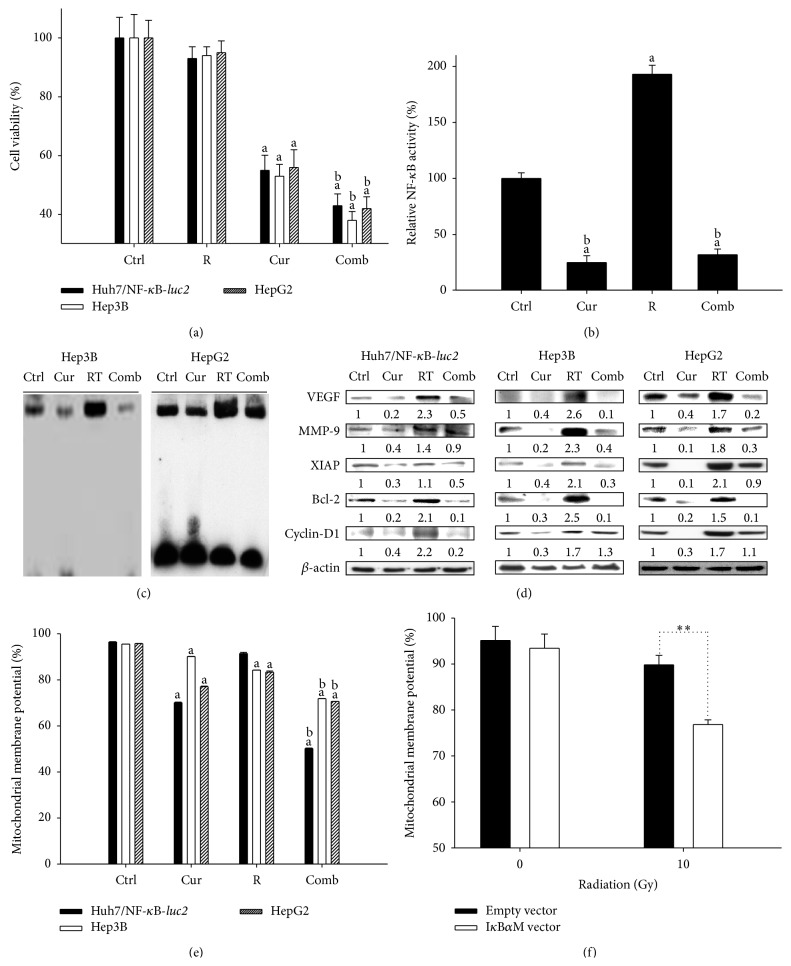
The effects of curcumin on radiation-induced cytotoxicity, NF-*κ*B activation, expressions of NF-*κ*B downstream effector proteins, and loss of MMP in HCC cells (Huh7/NF-*κ*B-*luc2*, Hep3B, and HepG2). Cells were treated with 30 *μ*M curcumin for 24 hr, 10 Gy irradiation, and curcumin plus radiation, respectively. (a) The cell viability was evaluated with MTT assay. a < 0.01 compared with that of the control; and b < 0.01 compared with that of curcumin group. (b) The NF-*κ*B activation was evaluated with IVIS50. a < 0.01 compared with that of the control; b < 0.01 compared with that of radiation group. (c) NF-*κ*B/DNA binding activity was determined by EMSA. (d) Expressions of NF-*κ*B downstream effectors proteins (i.e., VEGF, MMP-9, XIAP, Bcl-2, and cyclin-D1) were determined with Western blot. (e) The detection of MMP by flow cytometry. a < 0.01 compared with that of the control; b < 0.01 compared with those of radiation and curcumin groups. (f) Huh7/NF-*κ*B-*luc2* cells were transfected with either I*κ*B*α*M or empty vectors before irradiation. After irradiation, cells were incubated for 24 h and then assayed for MMP. ^*∗∗*^ *P* < 0.01. Ctrl: control, R: radiation, Cur: curcumin, and Comb: combination.

Curcumin combined with radiation shows the most effective cell killing compared with that of either agent in HCC cells (Huh7/NF-*κ*B-*luc2*, Hep3B, and HepG2) as shown in Figure 3(a). Curcumin suppresses radiation-induced NF-*κ*B activity assayed by bioluminescent imaging and EMSA, respectively, as shown in Figures 3(b) and 3(c). Expressions of NF-*κ*B downstream effector proteins, including VEGF, MMP-9, XIAP, Bcl-2, and cyclin-D1, increased by radiation are suppressed by curcumin (Figure 3(d)). Curcumin combined with radiation is shown to be the most effective to decrease MMP compared with that of either agent alone (Figure 3(e)). I*κ*B*α*M vector, a negative regulator of NF-*κ*B activation, was used to verify whether inhibition of NF-*κ*B activation can enhance radiation-induced apoptosis in Huh7/NF-*κ*B-*luc2* cells. I*κ*B*α*M vector transfection plus radiation was more effective to decrease MMP than empty vector transfection plus radiation (Figure 3(f)).

## 4. Discussion

Since the treatment outcome of radiotherapy for patients with hepatoma is not satisfactory, it is crucial to develop radiosensitizer to improve therapeutic efficacy. Our previous study has shown that curcumin could sensitize human oral cancer to radiation* in vitro* and* in vivo *[11]. Whether curcumin can enhance radiosensitivity of HCC, however, is ambiguous. In this study, three human HCC cell lines were used to evaluate the therapeutic efficacy of curcumin combined with radiation. The results show that curcumin enhances radiation-induced antitumor effects through suppression of radiation-increased NF-*κ*B activity and expressions of NF-*κ*B downstream effector proteins.

Radiation could trigger the activity of NF-*κ*B and its downstream effector proteins including those involved in antiapoptosis to acquire the ability of radioresistance in many human cancers [6]. Several studies show that inhibition of NF-*κ*B could enhance radiation-induced tumor growth inhibition by overcoming radioresistance while increasing radiosensitivity of tumor cells [11, 13, 14, 16]. I*κ*B*α*M vector, which constitutively expresses super repressor of NF-*κ*B, has been shown to augment radiation-induced cytotoxicity in human oral, lung, and cervical cancer cell lines [11, 21]. Here the effects of I*κ*B*α*M vector on radiation-induced the loss of mitochondrial membrane potential (ΔΨ_*m*_) in Huh7/NF-*κ*B-*luc2* cells were evaluated by flow cytometry. It was found that Huh7/NF-*κ*B-*luc2* cell line transfected with I*κ*B*α*M vector and combined with 10 Gy irradiation significantly increased the loss of MMP compared to those transfected with empty vector plus radiation as shown in Figure 3(f). The loss of MMP represents an early event in cellular apoptosis [22]. This result suggests that inhibition of NF-*κ*B activation enhances radiation-induced apoptosis. In this study, curcumin was found to suppress the radiation-induced NF-*κ*B activity and significantly enhance radiation-induced cytotoxicity and the loss of MMP.

Antiapoptotic proteins such as Bcl-2 and XIAP prevent loss of MMP and expression of caspase-3 activation leading to inhibition of apoptosis during cancer treatment [23, 24]. Some studies show that inhibition of NF-*κ*B activation negatively regulates the expressions of Bcl-2 and XIAP and results in the increase of radiation-induced apoptosis [11, 13, 14, 16]. Here we found that radiation-induced NF-*κ*B activity and expressions of antiapoptotic proteins such as Bcl-2 and XIAP were inhibited by curcumin in three human HCC cell lines as shown in Figures 3(c) and 3(d). VEGF and MMP-9, the main mediators of tumor angiogenesis and invasion, could be induced by radiation-triggered NF-*κ*B activity [11, 17, 18]. This study shows that expressions of VEGF and MMP-9 induced by radiation are suppressed by curcumin in three human HCC cell lines (Figure 3(d)). Taken together, we suggested that curcumin may enhance the therapeutic efficacy of radiation.

## 5. Conclusion

In conclusion, curcumin sensitizes human HCC cells to radiation via the suppression of radiation-induced NF-*κ*B activity and expressions of its downstream effector proteins. In addition, curcumin combined with radiation may have therapeutic potential for patients with HCC.

## References

[1] Parkin D. M. (2001). Global cancer statistics in the year 2000. *The Lancet Oncology*.

[2] Forner A., Hessheimer A. J., Isabel Real M., Bruix J. (2006). Treatment of hepatocellular carcinoma. *Critical Reviews in Oncology/Hematology*.

[3] Ren Z.-G., Zhao J.-D., Gu K. (2011). Three-dimensional conformal radiation therapy and intensity-modulated radiation therapy combined with transcatheter arterial chemoembolization for locally advanced hepatocellular carcinoma: an irradiation dose escalation study. *International Journal of Radiation Oncology Biology Physics*.

[4] Hawkins M. A., Dawson L. A. (2006). Radiation therapy for hepatocellular carcinoma: from palliation to cure. *Cancer*.

[5] Liu Y.-C., Chiang I.-T., Hsu F.-T., Hwang J.-J. (2012). Using NF-kappaB as a molecular target for theranostics in radiation oncology research. *Expert Review of Molecular Diagnostics*.

[6] Li F., Sethi G. (2010). Targeting transcription factor NF-*κ*B to overcome chemoresistance and radioresistance in cancer therapy. *Biochimica et Biophysica Acta—Reviews on Cancer*.

[7] Baud V., Karin M. (2009). Is NF-*κ*B a good target for cancer therapy? Hopes and pitfalls. *Nature Reviews Drug Discovery*.

[8] Li W., Tan D., Zenali M. J., Brown R. E. (2010). Constitutive activation of nuclear factor-kappa B (NF-*κ*B) signaling pathway in fibrolamellar hepatocellular carcinoma. *International Journal of Clinical and Experimental Pathology*.

[9] Guo R.-P., Zhong C., Shi M. (2006). Clinical value of apoptosis and angiogenesis factors in estimating the prognosis of hepatocellular carcinoma. *Journal of Cancer Research and Clinical Oncology*.

[10] Che Y., Ye F., Xu R. (2012). Co-expression of XIAP and cyclin D1 complex correlates with a poor prognosis in patients with hepatocellular carcinoma. *The American Journal of Pathology*.

[11] Chiang I.-T., Liu Y.-C., Hsu F.-T. (2014). Curcumin synergistically enhances the radiosensitivity of human oral squamous cell carcinoma via suppression of radiation-induced NF-kappaB activity. *Oncology Reports*.

[12] Yu W., Gu K., Yu Z. (2013). Sorafenib potentiates irradiation effect in hepatocellular carcinoma in vitro and in vivo. *Cancer Letters*.

[13] Kuo Y.-C., Lin W.-C., Chiang I.-T. (2012). Sorafenib sensitizes human colorectal carcinoma to radiation via suppression of NF-kappaB expression in vitro and in vivo. *Biomedicine and Pharmacotherapy*.

[14] Kunigal S., Lakka S. S., Joseph P., Estes N., Rao J. S. (2008). MMP-9 inhibition downregulates radiation-induced NF-*κ*B activity leading to apoptosis in breast tumors. *Clinical Cancer Research*.

[15] Shehzad A., Lee Y. S. (2013). Molecular mechanisms of curcumin action: signal transduction. *BioFactors*.

[16] Chendil D., Ranga R. S., Meigooni D., Sathishkumar S., Ahmed M. M. (2004). Curcumin confers radiosensitizing effect in prostate cancer cell line PC-3. *Oncogene*.

[17] Kunnumakkara A. B., Diagaradjane P., Guha S. (2008). Curcumin sensitizes human colorectal cancer xenografts in nude mice to *γ*-radiation by targeting nuclear factor-*κ*B-regulated gene products. *Clinical Cancer Research*.

[18] Chiang I.-T., Liu Y.-C., Wang W.-H. (2012). Sorafenib inhibits TPA-induced MMP-9 and VEGF expression via suppression of ERK/NF-kappaB pathway in hepatocellular carcinoma cells. *In Vivo*.

[19] Hsu F.-T., Liu Y. U.-C., Chiang I.-T. (2014). Sorafenib increases efficacy of vorinostat against human hepatocellular carcinoma through transduction inhibition of vorinostat-induced ERK/NF-*κ*B signaling. *International Journal of Oncology*.

[20] Wang W.-H., Chiang I.-T., Ding K. (2012). Curcumin-induced apoptosis in human hepatocellular carcinoma J5 cells: critical role of Ca^+2^-dependent pathway. *Evidence-Based Complementary and Alternative Medicine*.

[21] Lee C.-T., Mi Y. P., Dal R. K. (2009). Blockade of NF-*κ*B activation by I*κ*B*α* gene therapy enhances radiation sensitivity and abolishes acquired resistance to radiation. *Molecular Medicine Reports*.

[22] Ly J. D., Grubb D. R., Lawen A. (2003). The mitochondrial membrane potential (Δ*ψ*
_*m*_) in apoptosis; an update. *Apoptosis*.

[23] Shimizu S., Eguchi Y., Kamiike W. (1996). Bcl-2 blocks loss of mitochondrial membrane potential while ICE inhibitors act at a different step during inhibition of death induced by respiratory chain inhibitors. *Oncogene*.

[24] Bratton S. B., Lewis J., Butterworth M., Duckett C. S., Cohen G. M. (2002). XIAP inhibition of caspase-3 preserves its association with the Apaf-1 apoptosome and prevents CD95- and Bax-induced apoptosis. *Cell Death and Differentiation*.

